# Diamondites: evidence for a distinct tectono-thermal diamond-forming event beneath the Kaapvaal craton

**DOI:** 10.1007/s00410-019-1608-0

**Published:** 2019-08-19

**Authors:** S. Mikhail, F. M. McCubbin, F. E. Jenner, S. B. Shirey, D. Rumble, R. Bowden

**Affiliations:** 1 The School of Earth and Environmental Sciences, The University of St. Andrews, St. Andrews, Scotland, UK; 2 Department of Earth and Planetary Sciences, Institute of Meteoritics, University of New Mexico, Albuquerque, NM USA; 3 Present Address: NASA Johnson Space Centre, Houston, TX USA; 4 School of Physical Sciences, The Open University, Walton Hall, Milton Keynes, UK; 5 Department of Terrestrial Magnetism, Carnegie Institution of Washington, Washington, DC USA; 6 Geophysical Laboratory, Carnegie Institution of Washington, Washington, DC USA

**Keywords:** Diamond, Mantle petrology, Volatile elements, Stable isotopes, Garnet, Geochemistry, Cratonic mantle

## Abstract

The petrogenesis and relationship of diamondite to well-studied monocrystalline and fibrous diamonds are poorly understood yet would potentially reveal new aspects of how diamond-forming fluids are transported through the lithosphere and equilibrate with surrounding silicates. Of 22 silicate- and oxide-bearing diamondites investigated, most yielded garnet intergrowths (*n* = 15) with major element geochemistry (i.e. Ca–Cr) classifying these samples as low-Ca websteritic or eclogitic. The garnet REE patterns fit an equilibrium model suggesting the diamond-forming fluid shares an affinity with high-density fluids (HDF) observed in fibrous diamonds, specifically on the join between the saline–carbonate end-members. The δ^13^C values for the diamonds range from − 5.27 to − 22.48‰ (V-PDB) with δ^18^O values for websteritic garnets ranging from + 7.6 to + 5.9‰ (V-SMOW). The combined C–O stable isotope data support a model for a hydrothermally altered and organic carbon-bearing subducted crustal source(s) for the diamond- and garnet-forming media. The nitrogen aggregation states of the diamonds require that diamondite-formation event(s) pre-dates fibrous diamond-formation and post-dates most of the gem monocrystalline diamond-formation events at Orapa. The modelled fluid compositions responsible for the precipitation of diamondites match the fluid-poor and fluid-rich (fibrous) monocrystalline diamonds, where all grow from HDFs within the saline-silicic-carbonatitic ternary system. However, while the nature of the parental fluid(s) share a common lithophile element geochemical affinity, the origin(s) of the saline, silicic, and/or carbonatitic components of these HDFs do not always share a common origin. Therefore, it is wholly conceivable that the diamondites are evidence of a distinct and temporally unconstrained tectono-thermal diamond-forming event beneath the Kaapvaal craton.

**Electronic supplementary material:**

The online version of this article (10.1007/s00410-019-1608-0) contains supplementary material, which is available to authorized users.

## Introduction

The deep carbon cycle can be investigated on billion year timescales by studying the petrological and geochemical nature of mantle diamonds and their mineral/fluid inclusions and intergrowths. There are three main diamond types; monocrystalline, fibrous, and polycrystalline, where the latter are found as either framboids (i.e. boart) or diamondite (Shirey et al. 2013). The latter is a mixture of phases, predominantly comprised of diamond intergrown with silicates and oxides (also known as framesite; Kurat and Dobosi 2000). Diamondites are comprised of fine- to medium-grained diamond and garnet, with minor clinopyroxene and accessory phases, including but not limited to, rutile, sulphide, magnetite, cohenite, and Mg-chromite (see Jacob et al. 2014 for a review).

Despite being relatively understudied, these samples show some striking petrological and geochemical features which distinguish diamondites from their monocrystalline counterparts. For example: (1) a dominance of low-Ca eclogitic to websteritic garnets relative to garnets from monocrystalline diamonds (Dobosi and Kurat 2002, 2010; Gurney and Boyd 1982; Jacob et al. 2000, 2011, 2014; Kirkley et al. 1991; Stachel and Harris 2008), (2) diamond-garnet textures which imply silicate melt was present during diamond formation (Dobosi and Kurat 2002, 2010), (3) a total lack of olivine for all diamondites investigated (Jacob et al. 2014), and (4) low δ^13^C values and high δ^15^N values in samples with peridotitic garnets (Mikhail et al. 2013).

To place diamondite-formation into the wider context of diamond-formation and the deep carbon cycle, the major/trace element abundances and stable C–O isotope values for diamonds and syngenetic garnet intergrowths liberated from nodules of diamondite are used to model the fluid composition(s) and source(s) for the diamondite-forming fluids at Orapa, Botswana. These data are used to contrast diamondite-formation with fibrous and non-fibrous monocrystalline diamond-formation. This study examines long-standing notion that diamondite-formation represents a habitually ignored but volumetrically significant and distinct carbon-rich metasomatic (diamond-forming) event in the sub-continental lithospheric mantle (Jacob et al. 2000; Kurat and Dobosi 2000; Dobosi and Kurat 2002, 2010; Mikhail et al. 2013).

## Samples

**Fig. 1:**
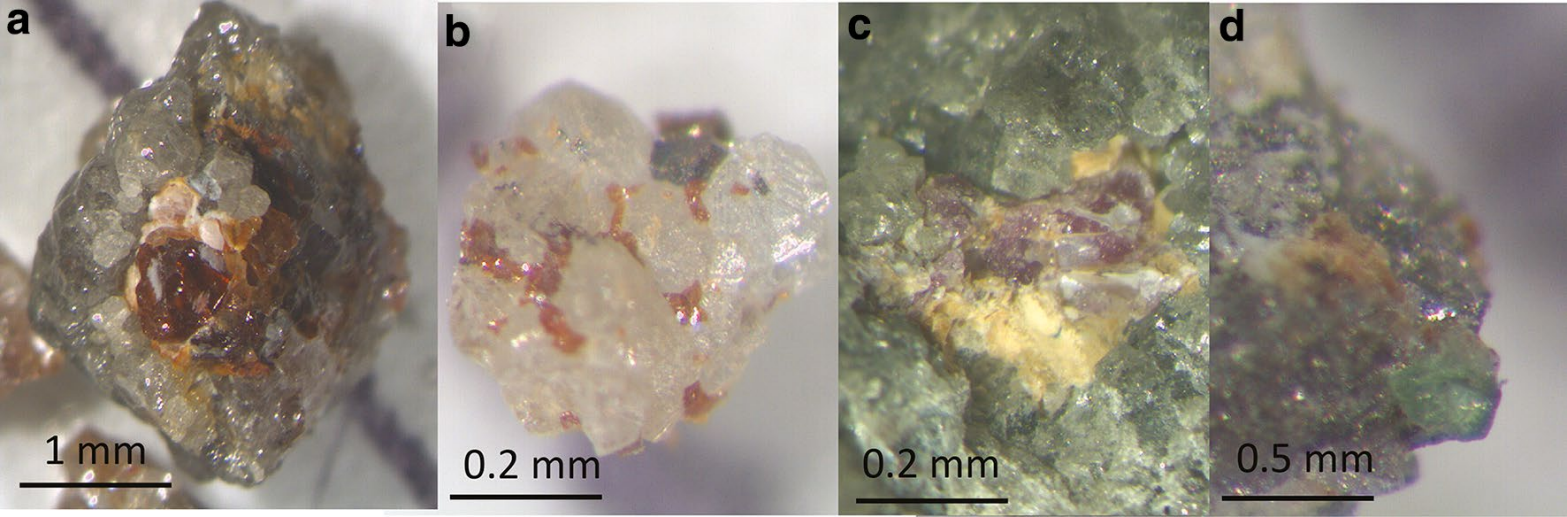
Photomicrographs for a selection of silicate diamondite samples from this study. The samples and silicate intergrowths shown are **a** ORF78 with an eclogitic garnet, **b** ORF145 with two websteritic garnets, **c** ORF53 with a peridotitic garnet, **d** sample26 with a peridotitic clinopyroxene

A total of 22 samples were selected from > 200 available for this study. The criteria for selection were that the samples contained observable diamond-silicate or diamond-oxide intergrowths and/or silicate inclusions in diamond. The samples come from the main Orapa kimberlite, Botswana, the largest of 23 pipes in the Orapa cluster in north-eastern Botswana, 250 km west of Francistown. The Orapa kimberlite was emplaced 93.1 Ma (Davis 1977) and lies on the western margin of the Kalahari Craton. The samples investigated here contained silicates (Fig. 1a–d) and oxides intergrown with polycrystalline diamond. The mining process at Orapa involves jaw-crushing the kimberlite, so these samples are pieces of larger nodules of diamondite that were broken during crushing. Examples of the fragmented nature of the samples and the intergrown nature of diamond and silicates are shown in Fig. 1a–d. Inclusions/intergrowths were liberated following cracking using a custom-made anvil, and conventional cross and straight peen hammer. The mineral phases were handpicked under a binocular microscope resulting in the recovery of a total of 15 garnets, 2 clinopyroxenes, 2 rutiles, 1 ilmenite, and 1 Mg-chromite (Table 1).

**Table 1:** Data table for the sample in this study showing the mineral intergrowths/inclusions picked from each sample, the major and minor element geochemistry of the intergrowths/inclusions, the δ^18^O value of garnets, and the δ^13^C values of the diamonds

Sample	ORF145a	ORF145b	ORF144a	ORF144b	ORF53a	ORF53b
Mineral	Garnet	Garnet	Garnet	Garnet	Garnet-W	Garnet-P
Paragenesis	Websteritic	Websteritic	Websteritic	Websteritic	Websteritic	Peridotitic
FeO	8.74	9.02	10.83	11.45	11.44	6.76
SiO_2_	42.29	42.49	41.65	41.59	42.12	41.91
TiO_2_	0.25	0.64	0.37	0.32	0.33	0.17
Al_2_O_3_	23.80	22.73	22.82	22.79	23.22	19.13
Cr_2_O_3_	0.28	0.29	0.15	0.14	0.14	5.62
MnO	0.31	0.30	0.32	0.33	0.36	0.35
MgO	20.24	20.85	19.07	17.79	18.02	20.77
NiO	0.01	0.02	0.00	0.01	0.01	0.01
CaO	4.66	3.95	4.43	4.79	4.79	5.07
Na_2_O	0.04	0.10	0.05	0.06	0.06	0.04
P_2_O_5_	0.03	0.02	0.02	0.02	0.02	0.02
Mg#	80.50	80.48	75.85	73.48	73.74	84.56
Total	100.66	100.41	99.72	99.29	100.53	99.85
*n* (analyses)	4	23	23	23	23	20
δ^18^O (‰)			+ 7.55			
δ^13^C (‰)	− 20.25	N/A	− 15.67	N/A	− 21.86	N/A

Sample	ORF119	ORF9	JJG897	ORF?	ORF32	ORF13
Mineral	Garnet	Garnet	Garnet	Garnet	Garnet	Garnet
Paragenesis	Websteritic	Websteritic	Websteritic	Websteritic	Websteritic	Websteritic
FeO	11.48	9.66	15.79	11.24	8.71	8.30
SiO_2_	41.17	42.03	40.31	41.62	42.25	42.19
TiO_2_	0.32	0.37	0.27	0.32	0.24	0.34
Al_2_O_3_	22.65	23.16	22.19	22.84	23.68	22.66
Cr_2_O_3_	0.14	0.21	0.09	0.13	0.27	0.60
MnO	0.36	0.31	0.40	0.37	0.30	0.31
MgO	18.16	19.36	15.71	18.11	20.15	21.57
NiO	0.03	0.02	0.06	0.01	0.00	0.02
CaO	4.79	4.53	3.79	4.86	4.66	3.40
Na_2_O	0.06	0.08	0.11	0.06	0.03	0.04
P_2_O_5_	0.02	0.02	0.03	0.03	0.03	0.02
Mg#	73.82	78.13	63.95	74.17	80.49	82.24
Total	99.19	99.75	98.75	99.59	100.31	99.48
*n* (analyses)	23	23	18	26	25	16
δ^18^O (‰)	+ 7.26					+ 5.96
δ^13^C (‰)	− 22.48	− 5.27	− 18.98	− 15.57	− 17.91	− 9.36

Sample	Sample26	ORF7	ORF7	ORF61	ORF78	ORF12
Mineral	Pyroxene	Pyroxene	Garnet	Garnet	Garnet	Ilmenite lamelle
Paragenesis	Peridotitic	Eclogitic	Eclogitic	Eclogitic	Eclogitic	Unknown
FeO	2.48	5.34	18.89	11.75	19.31	39.14
SiO_2_	54.22	54.44	39.94	41.09	38.81	0.00
TiO_2_	0.61	0.38	0.37	0.30	0.33	56.94
Al_2_O_3_	5.13	8.86	22.04	22.54	21.30	0.55
Cr_2_O_3_	1.32	0.05	0.06	0.23	0.07	0.10
MnO	0.07	0.05	0.39	0.24	0.38	0.01
MgO	13.74	9.45	9.64	13.44	9.74	2.80
NiO	0.03	0.01	0.01	0.02	0.02	0.06
CaO	16.28	14.36	8.75	10.42	8.54	0.00
Na_2_O	4.29	5.24	0.12	0.10	0.11	0.00
P_2_O_5_	0.01	0.00	0.01	0.01	0.02	0.00
Mg#	90.81	75.93	47.64	67.10	47.36	11.33
Total	98.18	98.19	100.24	100.14	98.62	99.61
*n* (analyses)	25	6	28	32	20	2
δ^18^O (‰)					+ 6.85	
δ^13^C (‰)			− 8.18	− 5.56	− 14.37	

Sample	ORF12	ORF41	ORF60	ORF49	ORF20	ORF114
Mineral	Rutile	Rutile	Chromite	Garnet	Garnet	Garnet
Paragenesis	Unknown	Unknown	Unknown	Websteritic	Websteritic	Websteritic
FeO	0.31	0.63	15.18	15.75	9.82	11.14
SiO2	0.00	0.06	0.05	40.18	42.28	41.62
TiO2	97.48	97.03	0.20	0.22	0.60	0.35
Al_2_O_3_	0.42	0.51	5.67	22.09	22.40	22.81
Cr_2_O_3_	0.11	0.29	63.14	0.08	0.13	0.14
MnO	0.00	0.07	0.40	0.38	0.38	0.34
MgO	0.00	0.07	12.07	15.50	20.30	18.43
NiO	0.02	0.18	0.12	0.02	0.02	0.01
CaO	0.00	0.03	0.01	4.21	3.83	4.61
Na_2_O	0.00	0.03	0.02	0.11	0.11	0.06
P_2_O_5_	0.00	0.02	0.00	0.03	0.02	0.02
Mg#	1.36	15.78	58.64	63.70	78.65	74.68
Total	98.36	98.92	96.87	98.56	99.90	99.52
*n* (analyses)	17	4	28	23	22	46
δ^18^O (‰)					+ 6.66	+ 7.55
δ^13^C (‰)				− 16.47	− 10.70	− 15.67

The value of n denotes the amount of EPMA analysis used to derive the mean the major and minor element geochemistry of each intergrowth/inclusion

## Methods

### Major and trace element data acquisition

Secondary electron images were produced using a JEOL JSM-6500F field emission scanning electron microscope at the Carnegie Institution of Washington (USA). Images were generated using an accelerating voltage of 15 kV on samples coated with iridium (~ 1 nm). Major and minor element compositions were determined by electron probe microanalysis (EPMA) using a JEOL 8200 electron microprobe housed within the Institute of Meteoritics at the University of New Mexico. Qualitative analysis was performed using both backscattered electron imaging and energy dispersive spectroscopy. Quantitative analyses were performed using wavelength dispersive spectrometers. An accelerating voltage of 15 kV and a nominal probe current of 40 nA was used during each analysis. We analysed for Si, Ti, Al, Cr, Fe, Mn, Mg, Ni, Ca, Na, and P. Si and Ca were standardized using a synthetic diopside crystal. Ti was standardized using Taylor rutile, and Fe was standardized using Taylor hematite (Taylor multi-element standard documentation. C.M. Taylor Company). Al was standardized using pyrope from Kakanui, New Zealand, and Cr was standardized using chromite from Tiebaghi Mine, New Caledonia (USNM 143968 and USNM 117075, respectively; Jarosewich et al. 1980). Mn was standardized on Taylor spessartine, and Ni was standardized using Taylor Ni-metal. Na was standardized on Albite from Amelia, Virginia. A natural fluorapatite from India (Ap020 from McCubbin et al. 2012) was used to standardize P. To reduce or eliminate electron beam damage, a 10 µm spot was used for both standardization and analysis of minerals in all samples. These data are reported in Table 1, and the full suite of data (including replicate analyses) are shown in supplementary Table S1.

Trace element abundances were determined by laser ablation (Photon Machines Analyte-193 laser ablation system, with a 193-nm wavelength excimer laser) inductively coupled plasma mass spectrometry (ICP-MS; Thermo iCAP-Q) at the Department of Terrestrial Magnetism, Carnegie Institution of Washington, USA. Data acquisition was performed with 20 s of background measurement, followed by 40 s of sample ablation using an ablation diameter of 238 µm and a repetition rate of 7 Hz. To minimize the potential for oxide production, the LA-ICP-MS system was tuned to low oxide production rates (ThO^+^/Th^+^ < 0.5%). Samples were analysed in batches of ~ 16, with multiple analyses of NIST SRM 612 (values given in Jenner and O’Neill 2012) used for external calibration of data, analysed at the beginning and end of each batch to allow corrections to be made for instrument drift. Data reduction was performed using the methods described in Jenner and O’Neill (2012) and using ^29^Si for internal calibration of data. Data for replicate analyses of reference material BCR-2G and comparisons with preferred values (presented in Jenner and O’Neill 2012, and references therein), included in each batch of analyses and the isotopes measured are presented in Supplementary Table 2. The relative standard deviation (RSD) of replicate analyses (*n* = 8) of BCR-2G, used as a measure of precision, are ≤ 5% for most elements, except for ^9^Be (5%), ^111^Cd (8%), ^115^In (8%), ^118^Sn (10%), ^121^Sb (7%), and ^182^W (6%). The higher RSD for these elements is attributed to their low elemental abundances. To ensure BCR-2G provided an accurate measure of LA-ICP-MS accuracy, the Department of Terrestrial Magnetism, Carnegie Institution of Washington, USA (DTM) chip of BCR-2G was analysed by LA-ICP-MS at the Research School of Earth Sciences, Australian National University using the LA-ICP-MS protocols described in Jenner and O’Neill (2012). Analyses of most elements are within 4% (typically ~ 2%) of the published values presented in Jenner and O’Neill (2012), demonstrating the suitability of BCR-2G as a reference material. Analyses of Cd (26% lower in DTM chip) are notably offset between the two chips of BCR-2G, demonstrating heterogeneity in the distribution of Cd between different chips of BCR-2G. Average analyses of BCR-2G analysed at DTM compared to the published values presented in Jenner and O’Neill (2012) and analyses of the DTM chip of BCR-2G are all within ≤ 9% of published values (typically better than 5% for most elements) demonstrating the accuracy of LA-ICP-MS analyses. The subtle offsets in analyses of BCR-2G (during the analytical session used to measure elemental abundances of trace elements in minerals for this contribution) compared to published values were used to perform a final normalization of data. The full data set is reported in supplementary Table S2.

### Quantifying the degree of nitrogen aggregation in the diamond

Infrared spectroscopic measurements were conducted at room temperature in transmittance mode on a JASCO IMV4000 Fourier Transform infrared spectrometer (FTIR) configured for the mid- to near-IR at the Geophysical Laboratory, Carnegie Institution of Washington, USA. Data were collected over the mid-IR (750–7800 cm^−1^) region using a standard light source, KBr beam splitter, and MCT detector. Approximately 256 scans were performed for each IR spectrum acquired at a resolution of 4 cm^−1^. Background spectra were collected under the same analytical conditions before each analysis and used to calculate absorbance by dividing each sample spectrum and then taking the base-10 logarithm. The IR spectra were deconvoluted using the excel version of the DiaMap software (Howell et al. 2012a, b). Uncertainties on each component are as follows; nitrogen content (± 10%) and aggregation state platelet intensity [*I*(*B*′)] (20%), platelet band position and height (± 1 cm^−1^), hydrogen-related band heights at 3107 and 1405 cm^−1^ (± 1 cm^−1^). While these uncertainties are very conservative, the reason for the large uncertainty on I(B´) is due to the exceptionally large platelet features that occur in some of the spectra and how variations in the baseline can significantly affect the result (Howell et al. 2012a, b). Several fragments of individual crystals were obtained from these samples by mechanically breaking small pieces off each sample. However, the transparency of the diamond crystals that form the diamondite can be highly variable, depending upon the concentration of included material (minerals and/or fluids; Mikhail et al. 2014a). As a result, only 7 samples yielded FTIR spectra of high enough quality to accurately determine the degree of nitrogen aggregation within the diamond.

### Stable isotope data acquisition

Oxygen isotope ratios were determined by heating samples with a 30 W Synrad CO_2_ laser in a 30-torr atmosphere of BrF_5_. Oxygen gas liberated by the fluorination of heated samples was transferred on molecular sieve (5A) frozen by liquid nitrogen to the inlet of a Thermo MAT252™ stable isotope ratio mass spectrometer at the Geophysical Laboratory, Carnegie Institution of Washington, USA. Analytical data are reported as per mil in relation to VSMOW (Vienna Standard Mean Ocean Water) by calibrating against reference materials NBS 28 and UWG-2 (Valley et al. 1995). Owing to the production of NF_3_ and CF_4_ during laser heating of diamond-garnet intergrowths in BrF_5_, the precision on δ^18^O values is ± 0.2‰.

Carbon isotopic data were made with a Thermo Scientific DeltaV_plus_™ gas-source mass spectrometer connected to a elementar Americas vario MICRO cube elemental analyser via a Conflo III interface in continuous-flow mode at the Geophysical Laboratory, Carnegie Institution of Washington, USA. The Conflo III interface also facilitates the introduction of the CO_2_ reference gas for the C isotope analyses. At least two blanks (or empty wells) were run between each sample to ensure there was no carry-over or a memory effect from un-combusted diamond/minerals. The elemental composition of the samples can account for complete combustion and no sample-to-sample contamination. In-house standards were analysed at regular intervals to normalize and correct these data (e.g., every 10–12 samples) and to monitor the accuracy and precision of the measured isotopic ratios and elemental compositions throughout the run. These in-house standards have been calibrated against international and other certified standards. All carbon isotopic values are reported relative to Pee Dee Belemnite (PDB) with the analytical error of ± 0.1‰. To demonstrate the applicability of the method employed here for the ^13^C/^12^C determinations of diamond, the results from our technique were compared to data from a single sample of known isotopic composition that has been previously analysed in two different laboratories using two different methods (Maruoka et al. 2004; Mikhail et al. 2013). The results of this test produced an average δ^13^C value for sample Dia053 of − 21.5 ± 0.17‰ (*n* = 6), which is within statistical error of the previous studies that produced an average δ^13^C value for sample Dia053 of − 20.9 ± 0.5‰ (*n* = 7; Maruoka et al. 2004; Mikhail et al. 2013).

## Results

### Petrography

**Fig. 2:**
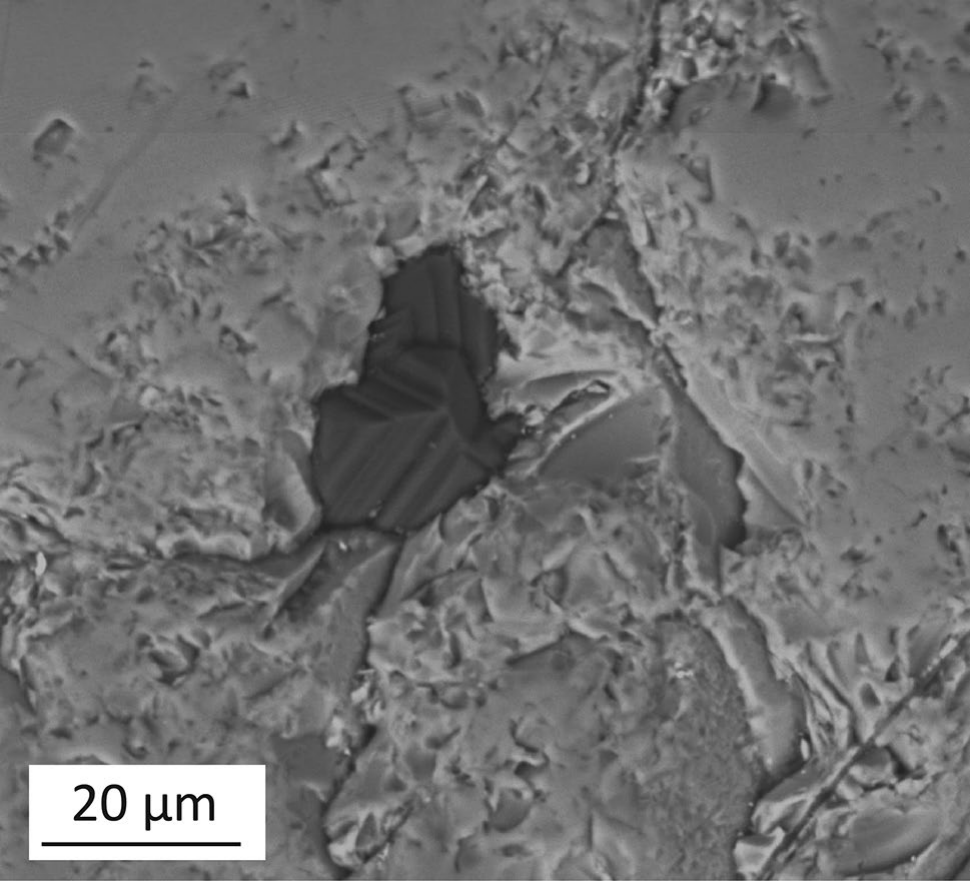
A secondary electron image showing a diamond inclusion within the peridotitic garnet from Fig. 1c

The samples show complex textural relationships with mutual intergrowths of diamond and garnet. This is consistent with previous observations of southern African diamondites (Kurat and Dobosi 2000; Dobosi and Kurat 2002, 2010; Rubanova et al. 2012), and that the garnet and diamond are syngenetic (Fig. 1b). For example, sample ORF53 (polished with alumina paste) contains a peridotitic garnet which is host to a diamond inclusion. The abundance of minerals intergrown together within these diamondites, in order of modal abundance, are pyrope garnet, clinopyroxene, rutile, ilmenite, and Mg-chromite (Table 1). Overall, the silicates and oxides are both interstitial and encapsulated within diamond (Figs. 1a–d, 2).

### Diamondite mineral chemistry and trace element abundances

**Fig. 3:**
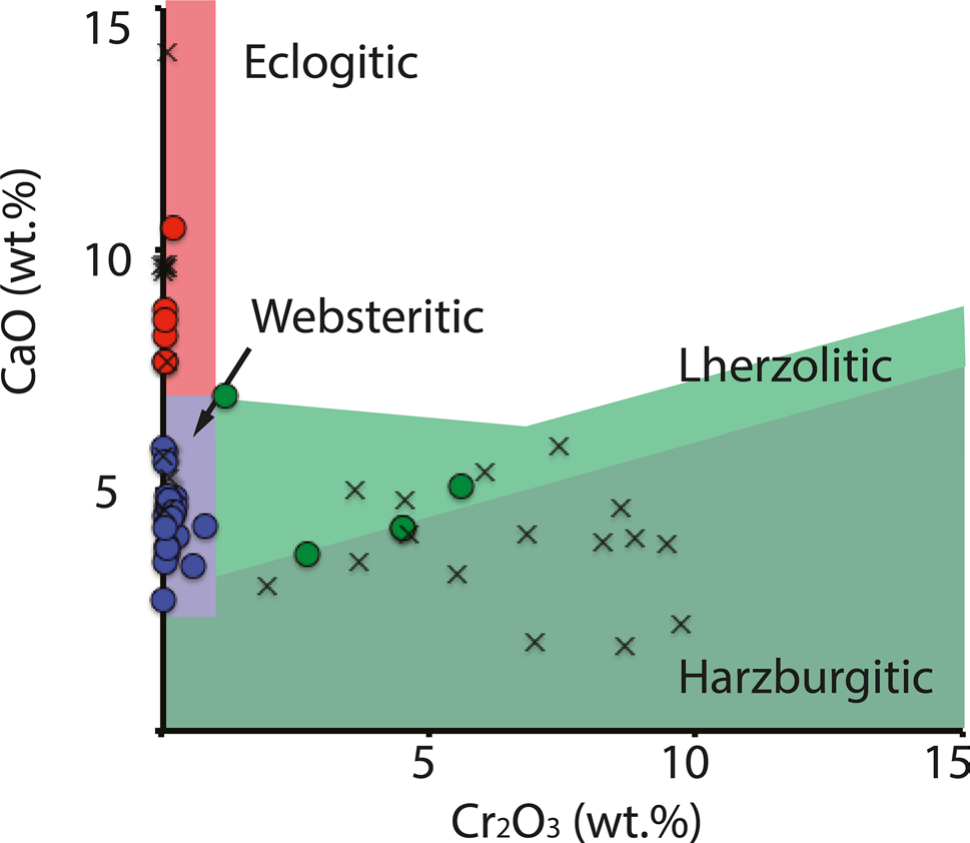
A Cr_2_O_3_ vs. CaO (wt %) garnet discrimination diagram with fields from Aulbach et al. (2002). The circle symbols are the garnets from the polycrystalline diamonds of this study (Table 1) and the literature (Gurney and Boyd 1982; McCandless et al. 1989) and the crosses are the data for the monocrystalline diamonds from Orapa (Deines et al. 1993; Stachel et al. 2004)

The average major element data for each sample are shown in Table 1, and the individual analyses from which the averages were calculated are provided in the supplementary material (Table S1). Of the 15 garnet-bearing samples, 13 are classed as websteritic, 3 are classed as eclogitic, and one is classed as peridotitic (using the Ca–Cr garnet discrimination diagram of Sobolev et al. 1973; Fig. 3). Sample ORF53 also shows a mixed paragenesis, where the largest garnet is peridotitic (lherzolitic) and a smaller garnet recovered is websteritic (Table 1) and sample ORF114 contains two chemically distinct websteritic garnets with differences for their CaO (4.4 vs 4.8 wt%), FeO (10.8 vs 11.4 wt%), and MgO (19.1 vs 17.8 wt%) contents (with Mg#’s from 75.8 vs 73.5; Table 1). Sample ORF145 also contained 2 websteritic garnets with no significant difference in major element geochemistry (Table 1). Using the Ca–Cr garnet discrimination diagram of Sobolev et al. (1973; Fig. 3), most garnets from diamondites classify as websteritic (or low-Ca eclogitic) (72%), whereas most garnets from monocrystalline diamonds classify as peridotitic (61%) (Fig. 6). The clinopyroxene inclusions/intergrowths are also dissimilar, where most clinopyroxenes from diamondites are peridotitic (> 90%), and most clinopyroxenes from monocrystalline diamonds are eclogitic (> 90%).

**Fig. 4:**
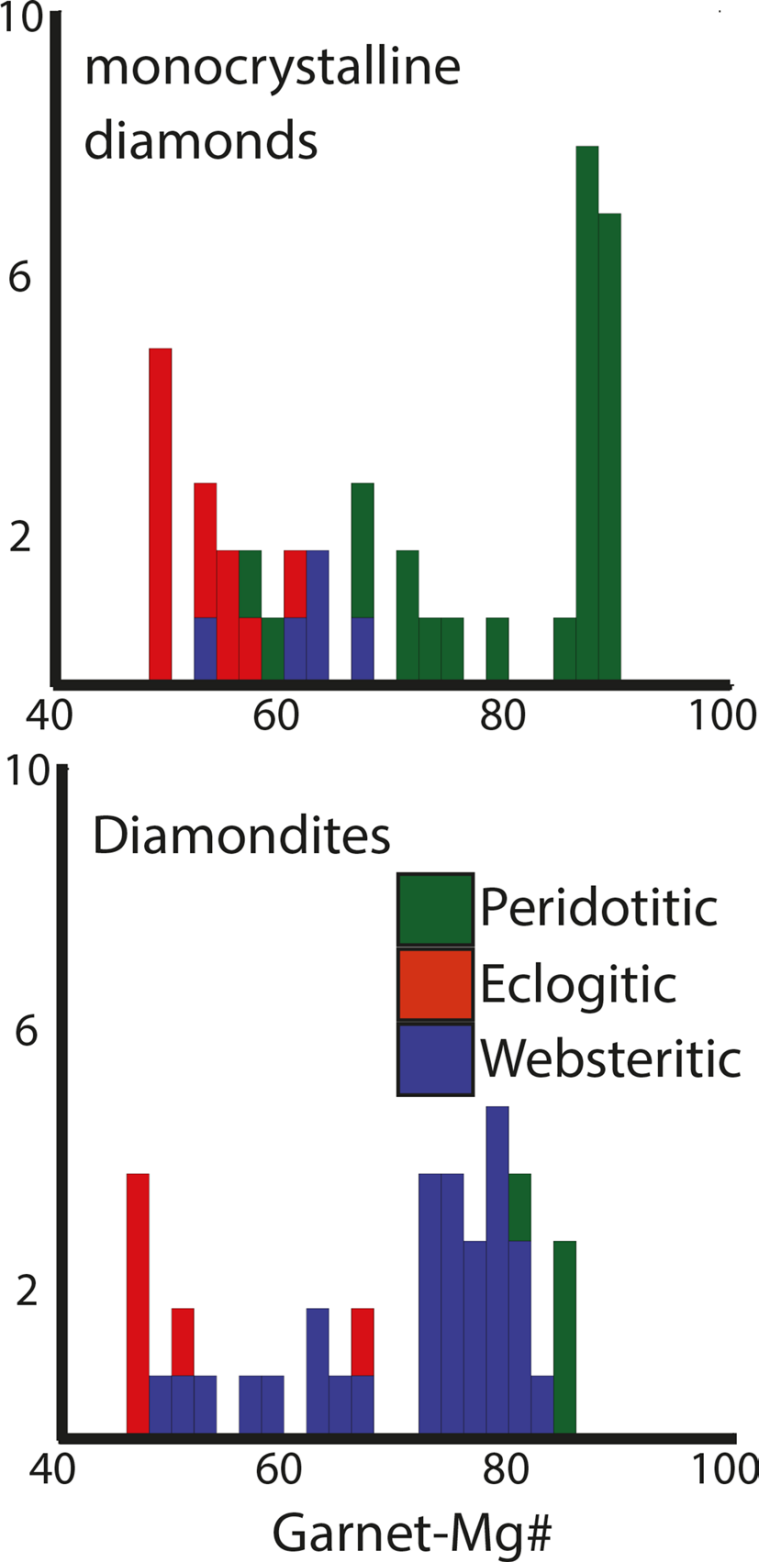
Histograms showing the Mg# for garnets from monocrystalline diamonds (top) (Deines et al. 1993; Stachel et al. 2004) and for garnets (bottom) (Gurney and Boyd 1982; McCandless et al. 1989; this study). Colours are the same as Fig. 3

The Mg numbers (Mg#) for the garnets vary among different samples over a large range (Mg#’s = 47–85). The websteritic garnets from diamondites show Mg#’s intermediate between the eclogitic and peridotitic garnets of the monocrystalline diamonds from Orapa (Fig. 4). Two eclogitic garnets (ORF7 and ORF61) and all the websteritic garnets are HREE-enriched, with a REE-patterns akin to eclogitic garnets from monocrystalline diamonds (Stachel et al. 2004). There are two pyroxene-types within these samples, one is eclogitic and the other peridotitic. The eclogitic clinopyroxene was hosted within sample ORF7 (which also contained an eclogitic garnet). Unfortunately, it is not known if these phases were in contact within the diamondite as the sample was opaque, and the clinopyroxenes were only observed after the mechanical disaggregation.

**Fig. 5:**
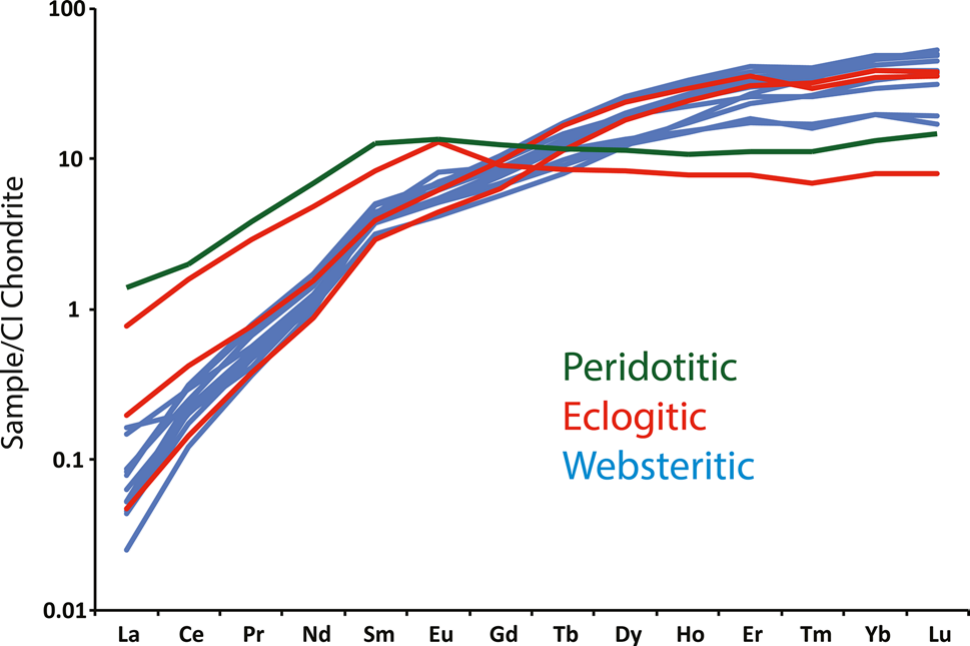
CI-chondrite normalized REE patterns for the average REE-abundances for the samples analysed in this study (CI-chondrite data from McDonough and Sun 1995). The green line corresponds to peridotitic, the red for eclogitic, and blue for websteritic

**Fig. 6:**
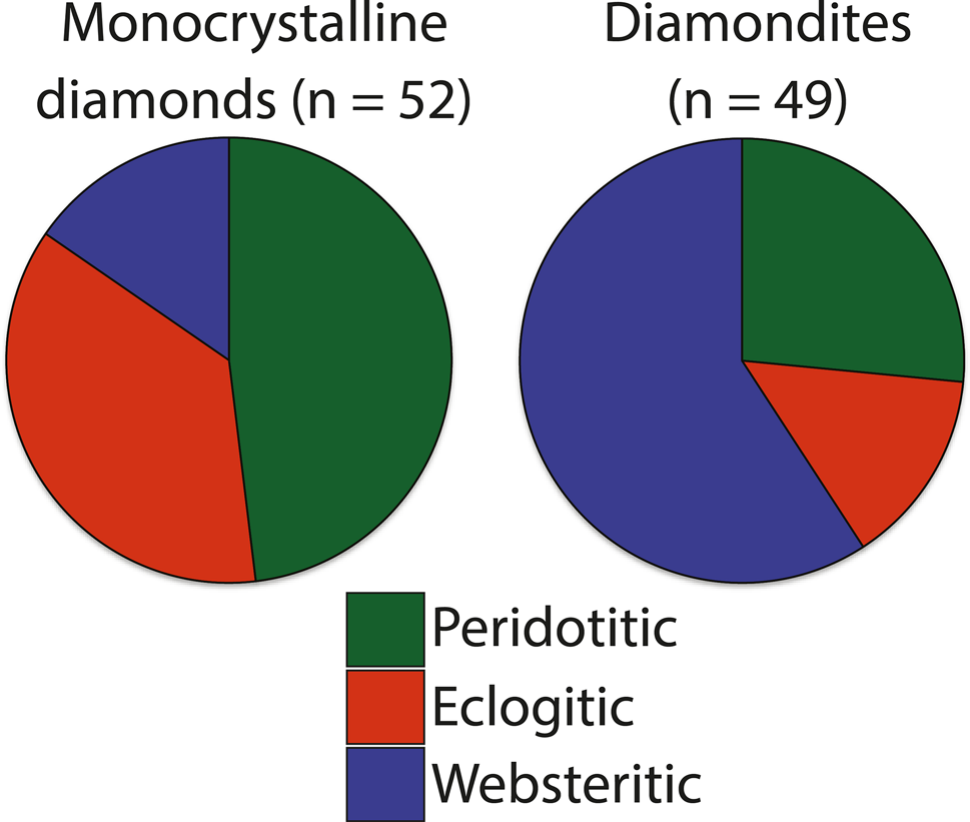
Pie charts showing the total abundance of peridotitic, eclogitic and websteritic silicate intergrowths and inclusions from diamondites (left) and monocrystalline diamonds (right) from the Orapa kimberlite, Botswana

Most garnets analysed here show distinct LREE-depletion and HREE-enrichment (Fig. 5a). None of these samples show significant trace element zonation based on multiple analysis of single garnets (Table S2). The average Rb/Sr for eclogitic-websteritic garnet inclusions (for diamondites) at Orapa is 0.300, the average Rb/Sr for eclogitic garnet inclusion from monocrystalline diamonds at Orapa being 0.039 (Timmerman et al. 2017; Richardson et al. 1990), with the highest Rb/Sr for garnets of monocrystalline diamonds showing an Rb/Sr of 0.23 (Timmerman et al. 2017). These data show the average websteritic garnet from diamondites and eclogitic garnets from monocrystalline diamonds show a considerable difference for their incompatible element enrichment, where the garnets from diamondites (this study) are more enriched, on average (e.g. Rb/Sr = 0.300 vs. 0.039) (Fig. 6).

### Stable isotope data

**Fig. 7:**
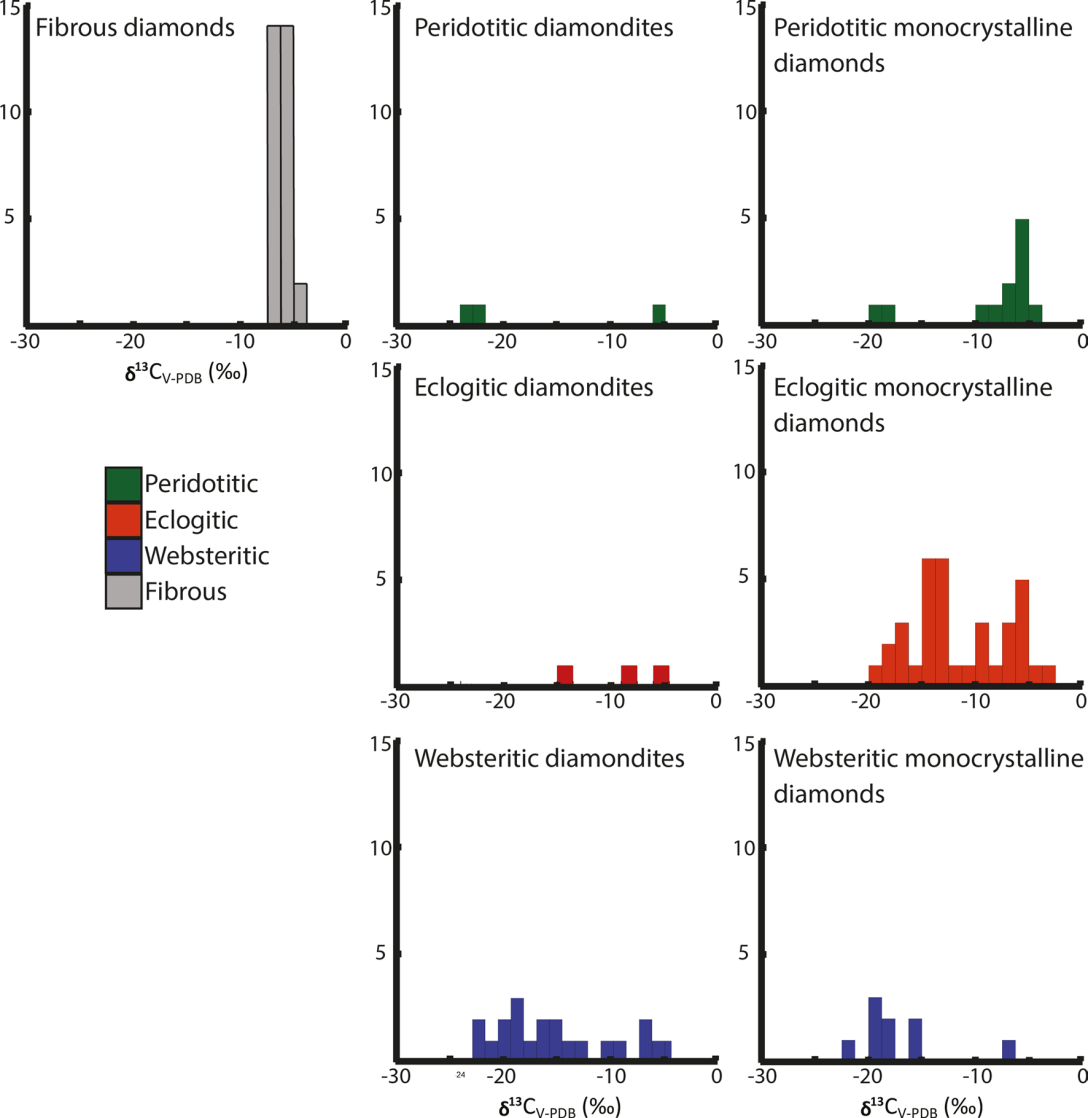
Histogram for the bulk δ^13^C values for diamondites, fibrous and monocrystalline diamonds as a function of mineral paragenesis. Fibrous and monocrystalline data from Deines et al. (1993), and diamondite data from this study and McCandless et al. (1989)

**Fig. 8:**
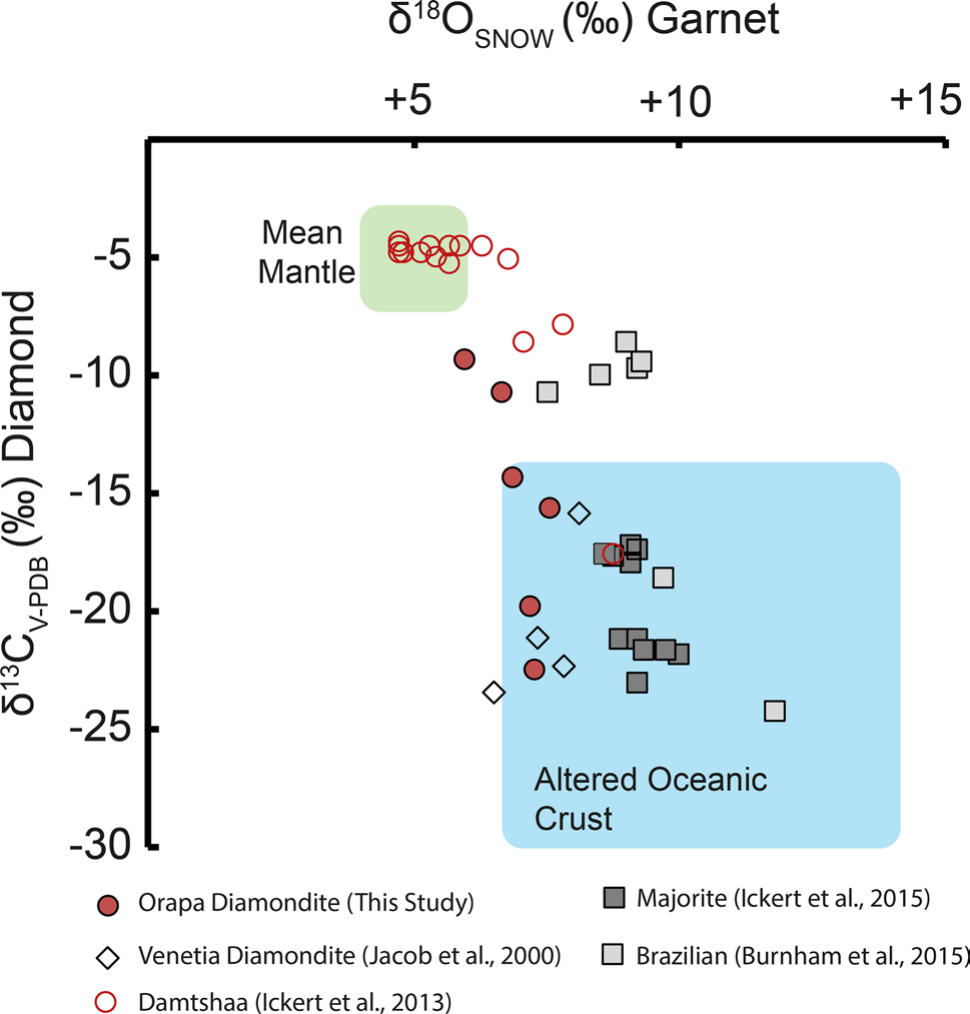
Variation diagram for the δ^18^O value of garnets vs. δ^13^C values of the diamonds from diamondites (this study) and monocrystalline diamonds from the Damtshaa kimberlite (Ickert et al. 2013). There are no coupled δ^18^O_garnet_ and δ^13^C_diamond_ data for samples from the main Orapa mine, but there are data for monocrystalline diamond-garnet couples from the Damtshaa kimberlite field, located roughly 20 km west of the main Orapa kimberlite field. The spatial proximity of the Damtshaa kimberlites to the Orapa kimberlites suggests that they sample the same mantle domain (e.g., Cartigny et al. 1999; Deines et al. 1993, 2009, Gurney and Boyd, 1982; Gurney et al. 1984; Shee and Gurney, 1979; Stachel et al. 2004; Viljoen et al. 1996; Ickert et al. 2013). Thus, these data are plotted for comparison. The eclogitic garnet-bearing monocrystalline diamonds from Damtshaa show a similar relationship in δ^18^O_garnet_ vs. δ^13^C_diamond_ space as the diamondites in this study

The range of δ^13^C values for the diamonds range from − 5.27 to − 22.48‰ (Fig. 7). Multiple analyses of different fragments from some of the same sample reveal a narrow internal variability for the δ^13^C values (average internal variability of ± 0.28‰), suggesting the mean values for multiple analyses of the samples represent the bulk value for the sample in question. The only peridotitic sample in the Orapa suite has a δ^13^C value of − 21.9‰. Although this is not uncommon for peridotitic diamondites (see Maruoka et al. 2004; Mikhail et al. 2013), it is extremely rare for peridotitic monocrystalline diamonds (see Deines 2002), but peridotitic monocrystalline diamonds from Orapa have previously been observed with low δ^13^C values (Deines et al. 1993). The δ^18^O values for websteritic garnets range from δ^18^O values of + 7.55 to + 5.96‰ (VSMOW), with an average value of + 6.91 (*σ *± 0.6)‰ and the sole eclogitic garnet analysed shows a δ^18^O value of + 6.9‰ (see Fig. 8).

### IR spectroscopy: lattice-bound hydrogen and nitrogen in the diamondites

The nitrogen abundance of the diamond liberated from these diamondites was determined by FTIR and show bulk sample average nitrogen abundances from 211 to 1235 at.ppm. However, the nitrogen abundances are heterogeneous within individual samples, with internal variabilities from 36 to 746 at.ppm, consistent with previous studies of diamondite (Mikhail et al. 2014b).

## Discussion

### Thermal and temporal constraints

Monocrystalline gem diamonds from Orapa (and neighbouring Letlhakane and Damtshaa) are evidence for a long and protracted record of carbonaceous metasomatism in the SCLM (Gurney et al. 2010). Diamond-hosted sulphide, clinopyroxene, and garnet inclusions yield eclogitic diamond formation-ages spanning a range of almost 3 billion years (e.g. at 0.1, 0.3, 1.0, 1.1, 1.7, 2.0, 2.3, 2.9, 3.0 Ga—Timmerman et al. 2017 and references therein). The only temporal data for fibrous diamonds are derived from nitrogen aggregation which require their formation shortly before emplacement in the colder upper crust by kimberlite volcanism at 91 Ma (Deines et al. 1993). However, there are no published models for the formation age of diamondites in the Orapa region.

In previous studies, generally contradictory formation ages for diamondites were obtained. Jacob et al. (2000) presented geochemical data supporting a young formation-age at Venetia (Jacob et al. 2000) whereas Mikhail et al. (2014b) presented nitrogen aggregation data supporting a relatively old formation-age for samples of unknown southern African provenance. However, in the absence of radiogenic isotope data, the timing of diamondite formation at Orapa can only be crudely evaluated using the geochemistry of garnets and the nitrogen aggregation states of the diamonds.

The observation of resolvable trace-element variability within diamondite-hosted garnets from Venetia (South Africa; Jacob et al. 2000) requires an emplacement age within a few thousands of years of kimberlite eruption because the time taken to diffusively equilibrate garnets with the mantle is in the order of 7 × 10^+4^ years at temperatures typical for diamond formation (> 1000 °C) (Jacob et al. 2000). The garnets in this study show no resolvable trace-element variability within single garnets (see Table S1). Therefore, the garnets in this study were either (1) not exposed to the mantle (i.e. by being fully-encapsulated by diamond), or (2) these garnets were exposed to the mantle and were simply not metasomatised by proto-kimberlitic fluids (unlikely). The geochemistry of the garnets in this study provides no evidence of a young formation age and no evidence for a metasomatic event around the time of emplacement at 91 Ma.

**Fig. 9:**
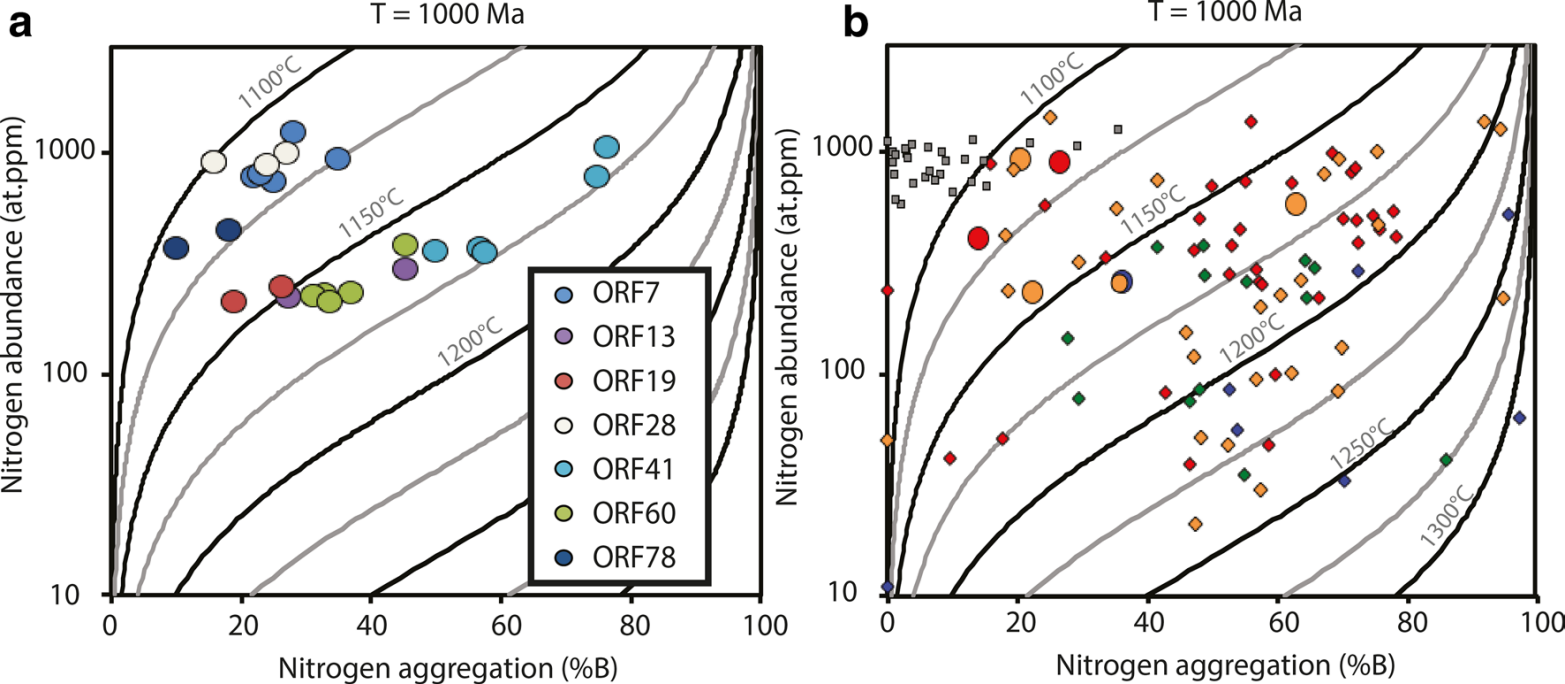
Variation diagrams for the degree of nitrogen aggregation vs. the nitrogen abundance (at.ppm) for the diamonds from diamondites in this study. The data on the left are for multiple analyses of single samples, and the data on the right show the averages for the diamondites in this study (circle symbols) alongside the fibrous diamonds (square symbols) and monocrystalline diamonds (diamond symbols) from Deines et al. (1993). The isotherms are calculated for residence times of 1000 Ma. Colours for the symbols on the right diagram the same as Fig. 3 using the DiaMap software (Howell et al. 2012a, b)

Nitrogen is the most common lattice-bound impurity in diamond, but its presence is metastable. As a result, nitrogen defects can develop over time from single nitrogen (C centres, Type Ib), to pairs of nitrogen atoms (A centres, Type IaA), to 4 nitrogen atoms tetrahedrally arranged about a vacancy (B centres, Type IaB; Evans and Qi 1982), in a process referred to as nitrogen aggregation. The first step in this process (C to A centre aggregation) occurs quite rapidly (< 1 Ma), while the second step (A to B centre) occurs much more slowly (over Ga). This A to B centre aggregation follows a second-order kinetics law (Chrenko et al. 1977) meaning nitrogen aggregation can be used to estimate either the average temperature of residence or the duration the diamond has resided in the mantle (assuming the other is known). Furthermore, platelets are planar interstitial carbon aggregates found on the {100} crystal planes, and these are the by-products of B centre formation and are prone to degradation during deformation and/or heating events (Woods 1986). In these samples platelet features are prevalent, but their intensity correlates with an increasing percentage of B-centres in the diamond. This means the abundance of B-centres show no evidence of degradation by deformation and/or heating (following the regularity plot of Woods 1986). The nitrogen aggregation state of these diamondites all show *B*-centres (advanced) of varying degree (10–76%B; Fig. 9a) and plot along calculated isotherms as a function of their nitrogen abundance (Fig. 9a). These give temperatures between 1100 and 1175 °C, if one assumes 1000 Ma lithospheric residence times. Note, the aggregation process is less controlled by time than it is by temperature, so nitrogen aggregation is more of a geothermometer than a temporal indicator. For example, the present calculation assumes 1000 Ma, however, if the temperature increased by a mere 25 °C, time is reduced by a factor of two (500 Ma).

The nitrogen aggregation state for single diamondite nodules from Orapa show relative homogeneity. This is important because it is known that single diamonds can grow episodically and reflect multiple growth events that can result in highly variable nitrogen aggregation states between different growth sectors formed at different geologic times (Boyd et al. 1987; Palot et al. 2013; Bulanova et al. 2014; Timmerman et al. 2017). For samples where ≥ 2 FTIR analysis were acquired, the data for single samples vary continuously along predicted isotherms (Fig. 9a). The observation of regular and internally continuous nitrogen aggregation data within these diamondites means that the nitrogen aggregation data for each sample can be accurately used to interpret their time–temperature history. The nitrogen aggregation data are fitted to a second-order kinetic model (Howell et al. 2012a, b) to ascertain information regarding the number, or depth, of the diamond-forming events (assuming different thermal regimes also reflect different mantle residence times). Thus, nitrogen aggregation data are used to provide an insight into the time–temperature histories between the three morphological diamond types sampled by the kimberlites of Orapa (Fig. 9b). All the Orapa diamondites show B-centres of varying degrees (10–76%B; Fig. 9a), and this requires several hundred million years of residence in the mantle prior to quenching due to emplacement in the crust at 91 Ma (Evans and Qi 1982; Taylor et al. 1996). The grouping of the diamond types at Orapa in terms of their nitrogen aggregation states (Fig. 9) can be viewed, with caution, as a rough guide to residence time (Mikhail et al. 2014c; Palot et al. 2013; Bulanova et al. 2014). There is a general relationship for diamond-type and nitrogen aggregation state at Orapa, whereby the fibrous samples show the least advanced nitrogen aggregation, followed by diamondites which do overlap with some gem-quality monocrystalline diamonds (Fig. 9). But most gem-quality monocrystalline diamonds display more advanced nitrogen aggregation for a given nitrogen abundance (Fig. 9). This implies that most of the gem-quality monocrystalline diamonds resided at high-temperatures for longer durations (i.e. they are older).

These nitrogen aggregation data (Fig. 9) require that diamondite-forming events pre-date fibrous diamond-formation and post-date most of the gem-quality monocrystalline diamond-forming episodes at Orapa. Thus, the nitrogen aggregation data offer the possibility that diamondite-formation at Orapa is the result of a younger tectono-thermal event than the formation event(s) for most gem-quality monocrystalline diamonds at Orapa.

### Origin of the diamond-forming carbon

The origin of diamond-forming carbon has been evaluated using the δ^13^C values of the diamond and the δ^18^O values of co-existing garnet. The diamondites and the fluid-poor monocrystalline diamonds show a larger range of δ^13^C values relative to their fibrous counterparts. The fibrous diamonds are derived from a source with a δ^13^C value consistently within the mean mantle value (Deines 2002; Fig. 7). The diamondites and the fluid-poor monocrystalline diamonds (of all parageneses) show evidence for a distinct component with ^13^C-depletion relative to the mean mantle (where mean mantle δ^13^C value = − 5 ± 3‰; Deines 2002). The ^13^C-depletion observed in the Orapa diamondites either reflects: (1) the ^13^C-depletion is the product of high-temperature carbon isotope fractionation (i.e. Cartigny et al. 1998; Smart et al. 2011; Mikhail et al. 2014b), or (2) the ^13^C-depletion reflects a crustal organic carbon source with lower ^13^C/^12^C ratio relative to the mantle (Boyd and Pillinger 1994). Carbon isotope data alone cannot resolve between these two contrasting explanations for ^13^C-depletion observed for the diamondites.

The origin of ^18^O-enriched or depleted oxygen in mantle samples is less dubious than ^13^C-depltion. This is because equilibrium stable isotope fractionation for ^18^O/^16^O at temperatures found in the mantle (> 800 °C) are very small and cannot explain a shift for a δ^18^O value > ± 1‰ (Chacko et al. 2001). Therefore, garnets with δ^18^O values > ± 1‰ outside of the mean mantle δ^18^O value of + 5.5 ± 0.5‰ (Mattey et al. 1994) are almost certainly sourced from material that was altered in a low-T environment (e.g. Earth’s crust) before being subducted back into the mantle (Jacob 2004; Schulze et al. 2004, 2013; Ickert et al. 2013, 2016; Burnham et al. 2015). The stable oxygen isotope data for the garnets from the diamondites in this study show ^18^O-enrichment with δ^18^O values of + 6.0 to + 7.6‰ (V-SMOW) with an average value of + 6.9 (± 0.6)‰. There are no δ^18^O data available for the fibrous diamonds, but the observed ^18^O-enrichment in the diamondite garnets is akin to what is observed for garnet inclusions in fluid-poor monocrystalline diamonds from Orapa and the neighbouring kimberlites of Damtshaa (Deines et al. 1991; Ickert et al. 2013; Fig. 8).

The combined C–O stable isotope data (Fig. 8) for the Orapa diamondites presented here are not consistent with a primary mantle origin, and support a model for a hydrothermally altered and organic carbon-bearing subducted crustal source(s) for the diamond- and garnet-forming media (Table 2).

**Table 2:** FTIR data for the samples in this study; nitrogen concentration (at.ppm), aggregation state (%*B*, i.e. proportion of the total nitrogen in the diamond in the form of *B* centres), height of the H band at 3107 cm^−1^, integrated area of the *B*′ platelet band (cm^−2^), and the wavenumber position of the *B*′ band maximum

Sample	Paragenesis	*N* ppm	%*B*	Nb	uB	*I*(*‘B*)	H@3107
ORF7_9	E	1235	28	345	4.340	116	0.584
ORF7_8	E	740	25	185	2.334	76	0.908
ORF7_7	E	943	35	330	4.155	55	0.455
ORF7_5	E	783	22	172	2.162	66	0.531
ORF7_1	E	802	23	184	2.322	96	0.409
Average		900	27				
ORF78_3	E	372	10	37	0.467	71	0.891
ORF78_1	E	444	18	80	1.005	102	0.661
Average		408	14				
ORF13_6	W	298	45	135	1.700	120	0.441
ORF13_2	W	224	27	61	0.766	86	1.321
Average		261	36				
ORF19_4	Ukn	249	26	65	0.820	25	0.310
ORF19_3	Ukn	214	19	41	0.510	19	0.161
Average		231	23				
ORF28_3	Ukn	997	27	268	3.375	59	0.626
ORF28_2	Ukn	876	24	209	2.634	67	0.314
ORF28_1	Ukn	915	16	144	1.817	59	0.018
ORF28	Ukn	915	16	144	1.819	59	0.018
Average		926	21				
ORF41_5	Ukn	358	50	179	2.252	117	4.774
ORF41_4	Ukn	368	57	209	2.632	139	4.892
ORF41_3	Ukn	352	58	202	2.544	116	4.626
ORF41_2	Ukn	1066	76	812	10.223	538	2.417
ORF41_1	Ukn	776	75	580	7.304	350	6.333
Average		584	63				
ORF60_6	Ukn	232	37	85	1.073	22	0.701
ORF60_4	Ukn	382	45	173	2.175	82	0.257
ORF60_3	Ukn	231	33	76	0.956	17	0.587
ORF60_2	Ukn	226	31	70	0.887	19	0.248
ORF60_1	Ukn	211	34	71	0.895	13	0.414
Average		256	36				

Each analysis for a given sample was performed on a different diamond fragment of the given sample. The IR spectra were deconvoluted using the excel version of the DiaMap software (Howell et al. 2012a, b). Uncertainties on each component are as such; nitrogen content and aggregation state—± 10%, platelet intensity [*I*(*B*′)]—± 20%, platelet band position and height—± 1 cm^−1^, hydrogen-related band heights at 3107 and 1405 cm^−1^—± 1 cm^−1^

### The petrogenesis of diamondites at Orapa

**Fig. 10:**
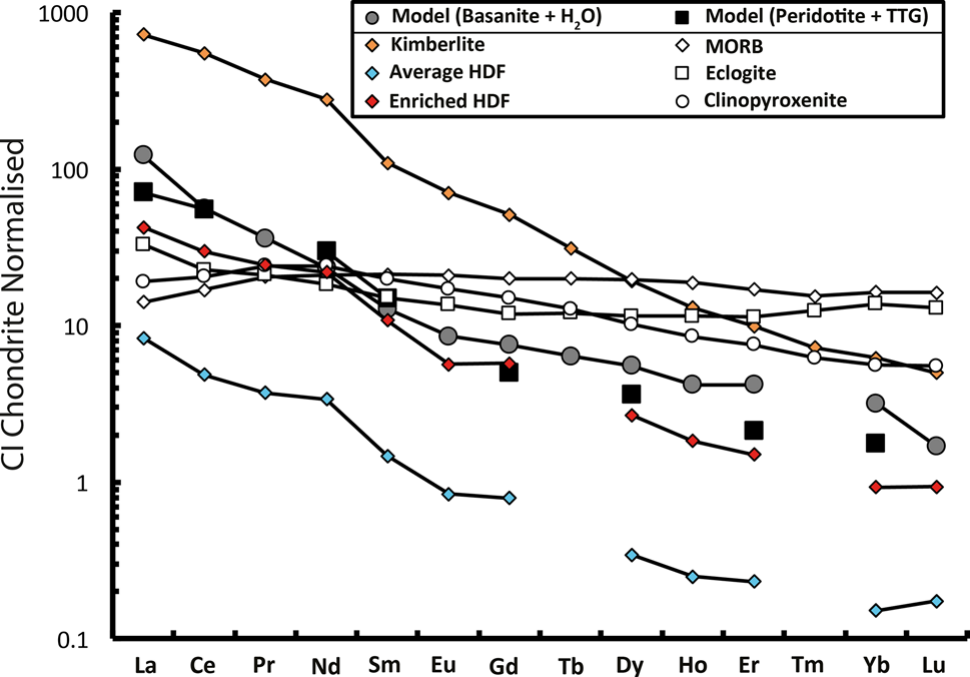
Calculated melts in equilibrium with garnet, using partitioning data for ca. 3 GPa and 1200 °C are from Rapp et al. (2010) (average of 3 experiments on peridotite + TTG) and Green et al. (2000) (basanite + H_2_O). Overlain with these model data are natural data for the pristine Lac de Gras kimberlites from Tappe et al. (2013), HDF data from Weiss et al. (2013), eclogitic melt data from the compilation of Aulbach and Jacob (2016), MORB data are from Arevalo and McDonough (2010), and clinopyroxenitic melt data from Perinelli et al. (2017) and Miao et al. (2017). Note, for the sake of clarity we have only reproduced the best fit data. We have not shown all the models attempted (i.e. carbonatite, tonalite, carbonaceous melt from sediments (pelite))

The websteritic garnets are by far the most abundant non-carbonaceous intergrowth in this sample suite, and so the nature of the diamondite-forming fluid at Orapa was investigated using the average REE geochemistry of the websteritic garnets. We have calculated melts in equilibrium with our garnets using D-values for various systems and compared the modelled melts with natural examples using the model detailed in Aulbach et al. (2013) but pioneered by Shimizu and Richardson (1987). The aim is to fingerprint the nature of the metasomatic agent in the SCLM by correlating which natural examples best fit our data (see Fig. 10 and caption). The modelled REE trends presented in Fig. 10 show the calculated equilibrium composition for the average REE abundances of melts using partitioning data for peridotite + TTG or basanite + H_2_O (partitioning data for ca. 3 GPa and 1200 °C from Green et al. (2000) and Rapp et al. (2010), respectively). The best fit model for the REE patterns and CI-normalised abundances is a HDF similar to those observed in fibrous diamonds by Weiss et al. (2013; on the join between the saline–carbonate end-members; Fig. 10). Because this population of diamondites are dominated by websteritic to low-Ca eclogitic garnets and with accessory intergrowths of rutile + ilmenite (Table 1), the favoured model is for the silicate source to share affinities with a high-Ti basaltic source (e.g. basanite).

The websteritic affinity of these garnets is interpreted to reflect mixing between peridotite and a websteritic to low-Ca eclogitic saline-carbonatitic HDF (akin to previous models for websteritic to clinopyroxenitic diamond-formation; Aulbach et al. 2002; Thomson et al. 2016; Kiseeva et al. 2013, 2016). Evidence for the role of a silicate melting event can be found in the enriched incompatible element geochemistry of garnets from diamondites relative to garnets from fluid-poor monocrystalline diamonds. For example, the garnets from diamondites show average Rb/Sr ratio of ~ 0.300, whereas garnets from monocrystalline diamonds show an average Rb/Sr ratio of ~ 0.039 (Richardson et al. 1990; Timmerman et al. 2017). The co-precipitation of diamond and a silicate melt is consistent with the textural relationship between the silicates and diamond, where diamond and silicates are intergrown (Figs. 1a–d, 2), and the mixed paragenesis (websteritic + peridotitic) observed in sample ORF53. The generation of an eclogitic melt during subduction can be explained by melting of carbonated oceanic crust (eclogite) at ca. 5.5 GPa and ca. 1100 °C in the presence of a free fluid-phase (with a high *a*_H2O_ or *a*_CO2_; Kessel et al. 2005; Dasgupta 2013). This is consistent with the nitrogen-aggregation derived temperature estimates of between 1100 and 1175 °C, which permit melting of a carbonated eclogite at pressures within the diamond stability field (Dasgupta et al. 2005).

The C–O stable isotope data strongly support the source of the carbon, and a significant portion of the silicate material, from hydrothermally altered and organic carbon-bearing subducted crustal sources (Fig. 8). The Orapa diamondites are the result of metasomatism of solid peridotite (SCLM) by a websteritic to low-Ca eclogitic saline-carbonatitic HDF derived from melting of a subducted basaltic protolith. This interpretation is supported by (1) the major element geochemistry of the garnet, specifically their low-Ca websteritic affinity (Fig. 3), (2) the trace-element geochemistry (Figs. 5, 10), and (3) the stable isotope geochemistry (Fig. 8).

Comparing the stable isotope and lithophile element geochemistry of diamondites with fibrous and fluid-poor monocrystalline diamonds (and their inclusions/intergrowths) from Orapa reveal that the diamond-forming carbon need not share a common origin (Fig. 7) despite sharing indistinguishable trace lithophile element geochemistry of the diamond-forming HDFs (Fig. 10).

## Conclusions

The cluster of kimberlites in the Orapa region sample all three main diamond types; monocrystalline, fibrous, and polycrystalline (diamondite). There are important differences for the petrogenesis of these three diamond types. The nitrogen in diamondites show more aggregated states than the fibrous diamonds but are less advanced than most fluid-poor monocrystalline diamonds. Thus, the nitrogen aggregation data offer the possibility that diamondite-forming event(s) at Orapa are younger than most fluid-poor monocrystalline diamonds but older than the fibrous diamond-forming events. The stable C–O isotope data for the Orapa diamondites support a model for a hydrothermally altered and organic carbon-bearing subducted crustal source(s) for the diamond- and garnet-forming media, which is similar to the volatile element source for some of the eclogitic fluid-poor monocrystalline samples, but distinct from the fibrous diamonds. Diamondites form as the result of rapid nucleation of polycrystalline diamond intergrown with silicates and oxide mineral phases (dominantly garnet). The fluids responsible for the precipitation of diamondites, and fluid-poor and fluid-rich (fibrous) monocrystalline diamonds share a geochemical affinity, where all grow from HDFs within the ternary saline-silicic-carbonatitic system. However, while the nature of the parental fluid(s) share a common lithophile element geochemical affinity, the origin(s) of the saline, silicic, and/or carbonatitic components of these HDFs do not always share a common origin.

Diamondites preserve the record of a distinct melting and metasomatic event in the SCLM initiated by the subduction of hydrothermally altered and organic carbon-bearing subducted crustal source(s). Therefore, it is wholly conceivable that the diamondites are evidence for a different and temporally unconstrained tectono-thermal diamond-forming event beneath the Kaapvaal craton.

## Electronic supplementary material

Below is the link to the electronic supplementary material.

The individual EPMA major element analysis on every inclusion/intergrowth analysed in this study (XLSX 124 kb)

The individual LA-ICP-MS acquired trace element abundance datum for every inclusion/intergrowth analysed in this study (XLSX 97 kb)
